# The reference shapes the signal: chrX genotype summaries and classifier sex disparity between GRCh38 and T2T-CHM13 callsets

**DOI:** 10.1186/s44342-026-00083-6

**Published:** 2026-10-16

**Authors:** R. Craig Stillwell

**Affiliations:** Independent Researcher, Hodgenville, KY 42748 USA

**Keywords:** Bioinformatics, Machine learning, Reference genome, Sex chromosomes, T2T-CHM13, Variant calling

## Abstract

Laboratories that move from GRCh38 to T2T-CHM13 take on a new callset as well as a new assembly. I asked whether the move changes sex-chromosome genotype summaries, and whether it changes how well classifiers trained on those summaries serve males and females. I compared the published 1000 Genomes GRCh38 and T2T-CHM13 callsets for 3,202 people. The two callsets come from different alignment, calling, phasing and filtering workflows, so every difference below belongs to the reference and its pipeline together. In women, the fraction of non-reference chrX genotypes that were heterozygous was 0.605 on GRCh38 and 0.644 on T2T. It fell in African-ancestry women and rose in the other four superpopulations. The rise came from fewer non-reference homozygous genotypes (− 7,757 per woman) and not from more heterozygous ones (− 1,504). Five classifier families were trained on six chrX summaries to predict superpopulation, and the male–female gap in balanced accuracy was compared between callsets. Women were classified better than men under both callsets. Logistic regression, random forest and XGBoost widened that gap on T2T, and the support vector machine and the perceptron narrowed it. After correction across all 20 tests, random forest, XGBoost and the support vector machine remained significant. Separating relatives did not remove the disagreement. Classifiers built from the first 500 common SNPs per chromosome in file order showed no change after correction. The gap measures model performance, not biological sex bias. Resolving the assembly from its callset requires processing the same reads through one pipeline against both references.

## Introduction

Many clinical and population genomics pipelines still align to GRCh38, whose quality across most of the genome remains high [1]. The T2T-CHM13 assembly closed the gaps that were left, and a complete reference improves the analysis of human genetic variation [2, 3]. Chromosome-scale haplotype-resolved assemblies built from long-read and Hi-C data [4] and draft human pangenomes [5] point the same way: from one linear sequence toward references that carry more of the variation in a population. Each step changes what a genotype means. A call is made against a particular sequence, and the public callsets that accompany each reference were produced with different aligners, callers and filters.

The sex chromosomes are a good place to look. T2T-CHM13 carries chrX but no Y chromosome, and a complete Y was assembled separately [3, 6]. The way a reference represents the sex chromosomes changes how many variants are called [7], and they are still left out of many association studies [8]. If a laboratory trains a classifier on genotype-derived features, a change of callset can alter how well that classifier works for men and for women. Differences in genomic prediction between groups are already a recognized problem [9].

I ask a narrow question here. When a laboratory swaps the published GRCh38 callset for the published T2T-CHM13 callset on the same people, what happens to chrX genotype summaries, and what happens to the male–female gap in the accuracy of classifiers trained on those summaries? The test bed is 3,202 people in the 1000 Genomes Project with both callsets available [10, 11]. Because the callsets were built by different workflows, the comparison cannot separate the assembly from its pipeline, and I treat it as a comparison of two callsets. I add controls that should not depend on chrX, and I check how much the answers depend on relatedness among the people sampled.

## Methods

### Cohort and data sources

I used the public 1000 Genomes Project callsets [10] for GRCh38 (the NYGC high-coverage, 30 ×, statistically phased panels [11]) and for T2T-CHM13v2.0 (the recalibrated PASS SNP and indel callset; variant calling for these 3,202 samples on T2T-CHM13 is described in [2]). The two files differ in assembly, in phasing, in post-calling processing, and in which sites they contain. I did not realign the same reads to both assemblies. The cohort is the 3,202 people present in both callsets: 1,603 females and 1,599 males in five continental superpopulations (Table 1).

**Table 1:** Cohort composition by superpopulation and sex

Superpopulation	Female (n)	Male (n)	Total (n)
AFR (African)	435	458	893
AMR (Admixed American)	265	225	490
EAS (East Asian)	293	292	585
EUR (European)	328	305	633
SAS (South Asian)	282	319	601
**Total**	**1,603**	**1,599**	**3,202**

All 3,202 people appear in the 1000 Genomes 20,130,606 pedigree file. Of these, 2,504 belong to the phase 3 integrated-call panel and 698 do not. In the full cohort 602 people have both parents in the cohort (597 parent pairs), and 2,003 people belong to one of 680 families that contribute more than one member. The phase 3 panel is not a set of unrelated individuals: 1,241 of its members share a family with another member.

Only public, de-identified data were analyzed. No new human subjects were enrolled, and institutional review was not required for this secondary analysis.

### Per-sample chrX and chr22 summaries

Per-sample genotype counts for the whole of chrX and, as an autosomal comparison, chr22 came from bcftools stats -s—[12] (PSC records). For SNPs, bcftools reports heterozygous genotypes (nHet), non-reference homozygous genotypes (nNonRefHom), haploid reference and alternative calls (nHapRef, nHapAlt, used for males on chrX), and missing genotypes (nMissing). The stored tables hold nHet, nNonRefHom, nHapRef, nHapAlt and nMissing. They do not hold the count of reference-homozygous genotypes.

Two consequences follow. The stored “heterozygosity rate” is the heterozygous fraction of non-reference genotypes in a diploid sample, nHet/(nHet + nNonRefHom), and I call it the heterozygous fraction. In a male, whose haploid calls enter the denominator, the same stored ratio is a different quantity and is not compared with the female value. The stored “call rate” is (nHet + nNonRefHom + nHapRef + nHapAlt) divided by the same sum plus nMissing, so it leaves out diploid reference-homozygous genotypes and is not a conventional call rate. I therefore report genotype counts, which are unambiguous, alongside these ratios.

### Statistical analysis of the summaries

Paired comparisons across callsets used Wilcoxon signed-rank tests [13], and mean paired differences used bootstrap 95% confidence intervals (2,000 resamples) [14]. For women on chrX I computed the paired change in the heterozygous fraction, in nHet and in nNonRefHom within each superpopulation, each with its own bootstrap interval. A Kruskal–Wallis test [15] compared the paired changes in the heterozygous fraction among superpopulations. The same paired contrasts were computed for chr22, separately in women and men.

### Panel-battery protocol

#### Estimand

For callset *r* and classifier *m*, the sex gap is *g* = BA(male) − BA(female), where BA is balanced accuracy (the mean of per-class recall) on held-out test people. The estimand, ΔΔ, is *g* on GRCh38 minus *g* on T2T. In Panel A the gap is negative under both callsets, because women are classified better than men. A positive ΔΔ therefore means the female advantage is larger on T2T, and a negative ΔΔ means it is smaller. ΔΔ is a difference in classifier performance on held-out samples.

#### Panels

**Table:** 

Panel	Task	Features	Role in the audit
A	Superpopulation classification	six chrX summaries	Features built from the chrX counts that differ between callsets
B	Sex classification	six chrX summaries	Sex is nearly separable from these features
C	Superpopulation classification	Common SNPs, 500 per chromosome × 23, first in VCF order	Control under this selection rule
D	Superpopulation classification	Common SNPs, chr1–22 only, first 500 per chromosome in VCF order	Control under this selection rule

The six chrX summaries are the stored heterozygosity ratio, the stored call rate, the stored homozygous fraction, nHet, the number of called genotypes (nHet + nNonRefHom + nHapRef + nHapAlt), and the number of non-reference genotypes (nHet + nHapAlt). Because diploid and haploid genotypes enter these quantities differently, several of them mean something different in men and in women. The stored homozygous fraction is zero in every woman and about 0.96 in men, and the called-genotype count is about 130,000 in women and 2.7 million in men. Panel B therefore separates the sexes almost trivially, and Panel A asks what an ancestry classifier does with summaries whose construction depends on sex.

Panels C and D used the first 500 SNPs per chromosome in VCF order with minor-allele frequency ≥ 0.01 and call rate ≥ 0.95, selected separately on each callset. The rule is reproducible but is not a random sample of common SNPs, is not LD-pruned, and does not match loci across assemblies. Because the rule takes the start of each chromosome, the chrX columns come from the pseudoautosomal region (GRCh38 positions 13,367–278,204; T2T 1,800–62,228). Genotypes were coded as the sum of the two allele indices. That equals the alternate-allele count at biallelic sites only. The GRCh38 matrix contains only 0, 1 and 2, but the T2T matrix also contains 3 and 4 (157,000 of 36.8 million cells, in 3,363 of 11,500 SNPs), where multiallelic records carry allele indices above 1. I refit Panel C with the T2T values capped at 2, which is exact for 1|2 and 2|2 genotypes and leaves 0|2 genotypes miscoded as 2.

### Classifiers and evaluation

Five families were fit: logistic regression, random forest, XGBoost [16], a radial-kernel support vector machine, and a multilayer perceptron [17]. Twenty stratified splits (70/15/15 train/validation/test by superpopulation × sex; seed 20,260,222 + repeat) gave about 2,241 training and 481 test people per repeat. Genotypes were dosage-encoded and missing values were filled with the training-set median. Logistic regression, the support vector machine and the perceptron were scaled inside the pipeline with a standard scaler (centering off, with_mean = False) fit on the training fold; random forest and XGBoost were not scaled. Hyperparameters were fixed across callsets within each panel.

Relatedness was examined by refitting Panel A three ways with the same models, features and splits: on the 2,504 phase 3 panel members; on all 3,202 people with every member of a pedigree family assigned to the same split; and on one person per family (*n* = 1,879), choosing a person recorded as unrelated when the family had one. Panels C and D were not refit with relatives separated.

#### Confirmatory tiers

For each model I computed bootstrap 95% confidence intervals (5,000 resamples) and a paired sign-permutation p-value (5,000 permutations) over the 20 splits. Holm–Bonferroni correction [18] was applied across the five models within a panel, and again across all 20 panel × model tests. Because the splits share people, these intervals and p-values describe how stable ΔΔ is across random splits and do not measure sampling error in a population. The pre-specified tier rule asked for directional consistency, one interval excluding zero, one Holm-significant model, and an effect of at least 0.01. It scored direction from the median ΔΔ across models, which can pass when models disagree. I therefore report the sign of every model and assign no tier to a panel in which the signs differ.

### Robustness

Panel C was checked with label permutation, leave-one-superpopulation-out validation, and exact sex-balance downsampling (*n* = 2,150). These checks do not block on family.

### Software and reproducibility

The original analyses ran under Python 3.12.13 with scikit-learn 1.8.0, XGBoost 3.0.3 and SciPy 1.16.0. The revision analyses (relatedness refits, capped Panel C refit, count decomposition and figures) ran under Python 3.13.1 with scikit-learn 1.8.0, XGBoost 3.3.0 and SciPy 1.14.1, together with NumPy and pandas. Code and the original results are at https://github.com/rstil2/reference-genome-sex-bias. The count decomposition, the stratified contrasts, the relatedness refits, the family-wide Holm correction and the figures are produced by revision_count_decomposition.py, revision_stratified_contrasts.py, revision_panelA_relatedness.py, revision_family_holm.py and revision_figures.py. They read the stored per-sample tables and the 1000 Genomes 20,130,606 pedigree file.

## Results

### Heterozygous fraction and genotype counts in women

On chrX the heterozygous fraction in women was 0.6052 (SD 0.0616) on the GRCh38 callset and 0.6443 (SD 0.0550) on the T2T callset. The paired difference was + 0.0391 (95% CI [+ 0.0375, + 0.0405]; Wilcoxon *p* = 5.23 × 10^−200^; 6.5% of the GRCh38 mean; Fig. 1a). The change is not the same in every superpopulation (Table 2; Fig. 1b). It is − 0.0055 in African-ancestry women (95% CI [− 0.0063, − 0.0048]; *n* = 435) and positive in the other four groups: + 0.0512 in AMR ([+ 0.0492, + 0.0533]; *n* = 265), + 0.0438 in EAS ([+ 0.0428, + 0.0448]; n = 293), + 0.0717 in EUR ([+ 0.0705, + 0.0730]; *n* = 328) and + 0.0538 in SAS ([+ 0.0525, + 0.0550]; *n* = 282). Each within-group Wilcoxon test rejects zero (all *p* < 10^−40^), and the five paired changes differ from one another (Kruskal–Wallis H = 1222.2, *p* = 2.49 × 10^−263^).

**Fig. 1:**
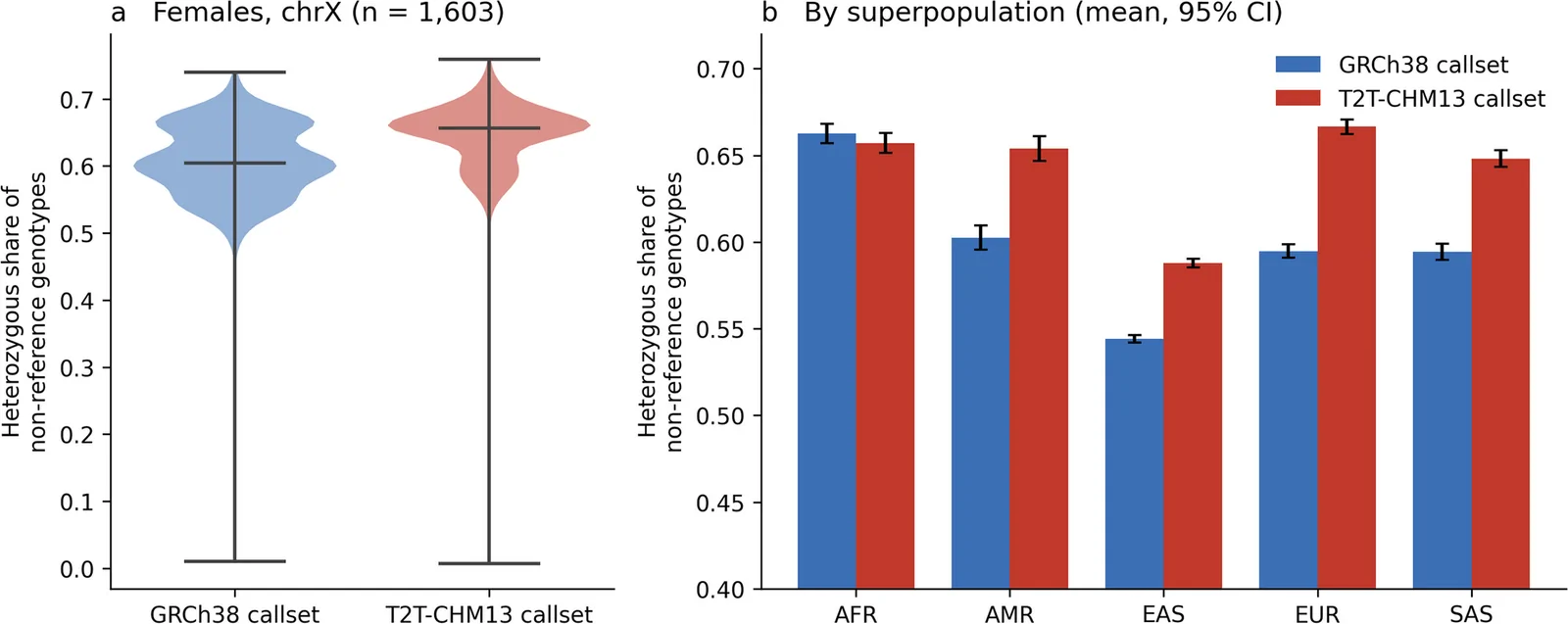
Heterozygous fraction of non-reference chrX genotypes in women. **a** Distribution on each callset. **b** Mean and 95% CI by superpopulation

**Table 2:** Women, chrX: heterozygous fraction and paired change in genotype counts (T2T minus GRCh38)

Superpopulation	n	Fraction, GRCh38	Fraction, T2T	Δ fraction	Δ nHet	Δ nNonRefHom
AFR	435	0.6627	0.6571	− 0.0055	− 2,767	+ 19
AMR	265	0.6026	0.6538	+ 0.0512	− 1,555	− 10,181
EAS	293	0.5442	0.5880	+ 0.0438	− 544	− 8,870
EUR	328	0.5949	0.6665	+ 0.0717	− 1,106	− 12,860
SAS	282	0.5944	0.6481	+ 0.0538	− 966	− 10,385
All women	1,603	0.6052	0.6443	+ 0.0391	− 1,504	− 7,757

The counts behind the fraction show where the change comes from (Fig. 2). Across all women, nHet fell by 1,504 per person (95% CI [− 1,551, − 1,454]) and nNonRefHom fell by 7,757 ([− 8,014, − 7,503]). The fraction rose because non-reference homozygous genotypes dropped by more than heterozygous ones. The pattern is not uniform. In AMR, EAS, EUR and SAS, nNonRefHom fell by 8,870 to 12,860 per woman. In African-ancestry women it did not change (+ 19; 95% CI [− 145, + 185]), while nHet fell by 2,767, so the fraction went down. Heterozygous genotypes did not increase in any group.

**Fig. 2:**
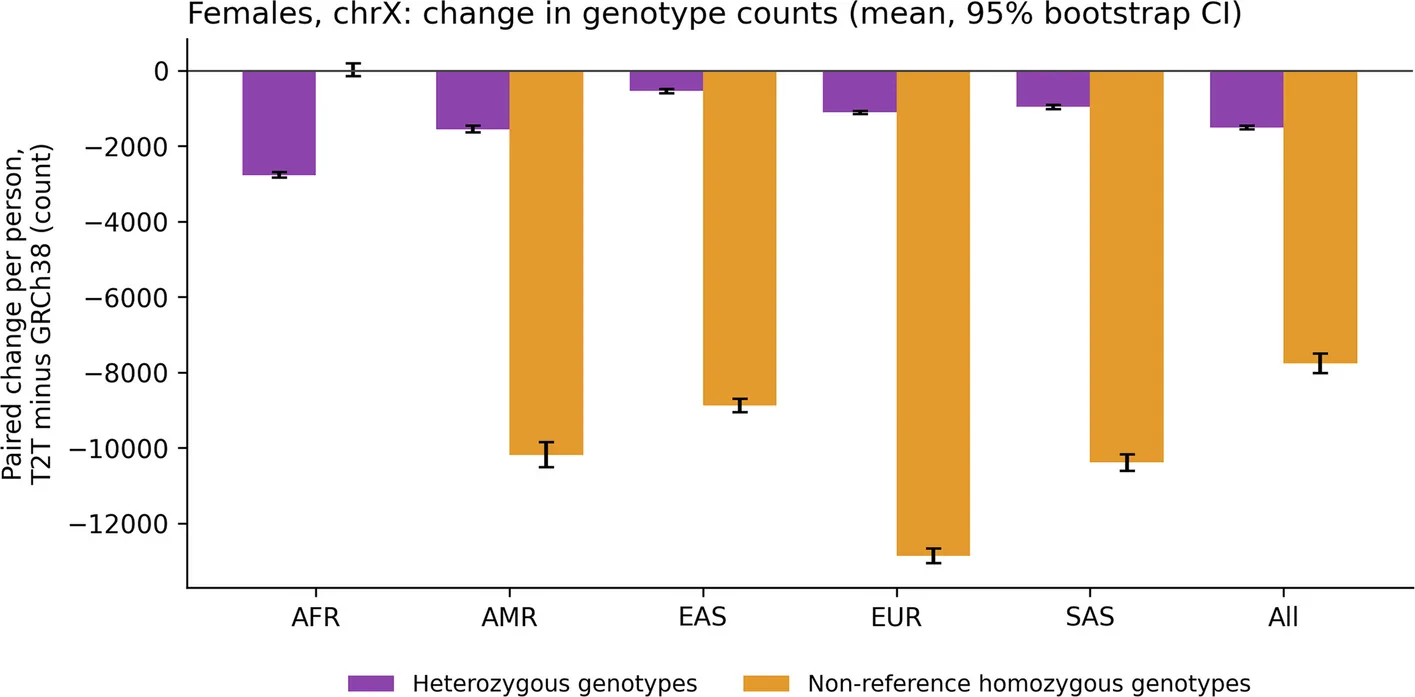
Paired change in chrX genotype counts in women (T2T minus GRCh38), by superpopulation. Mean and 95% bootstrap CI

A fall in non-reference homozygotes would follow if a site’s alternative allele on one assembly is the reference allele on the other, and differences in site lists and filtering could produce a similar pattern. I tested neither.

### Men, missing genotypes, and chr22

Men differ from women in how the counts are built. Men carried 4,690 heterozygous genotypes on the GRCh38 callset and 4,851 on the T2T callset (paired change + 161; 95% CI [+ 89, + 198]), against about 79,900 and 78,400 in women. Men’s haploid reference calls rose from 2.62 million to 4.32 million per person, so the T2T chrX file holds far more records per man than the GRCh38 file. The GRCh38 callset has no missing genotype in any person on either chromosome, which is consistent with a statistically phased panel; I could not check this against the VCF records, which are not stored with the analysis. The T2T callset has 10,752 missing genotypes per woman and 15,718 per man on chrX (Fig. 3).

**Fig. 3:**
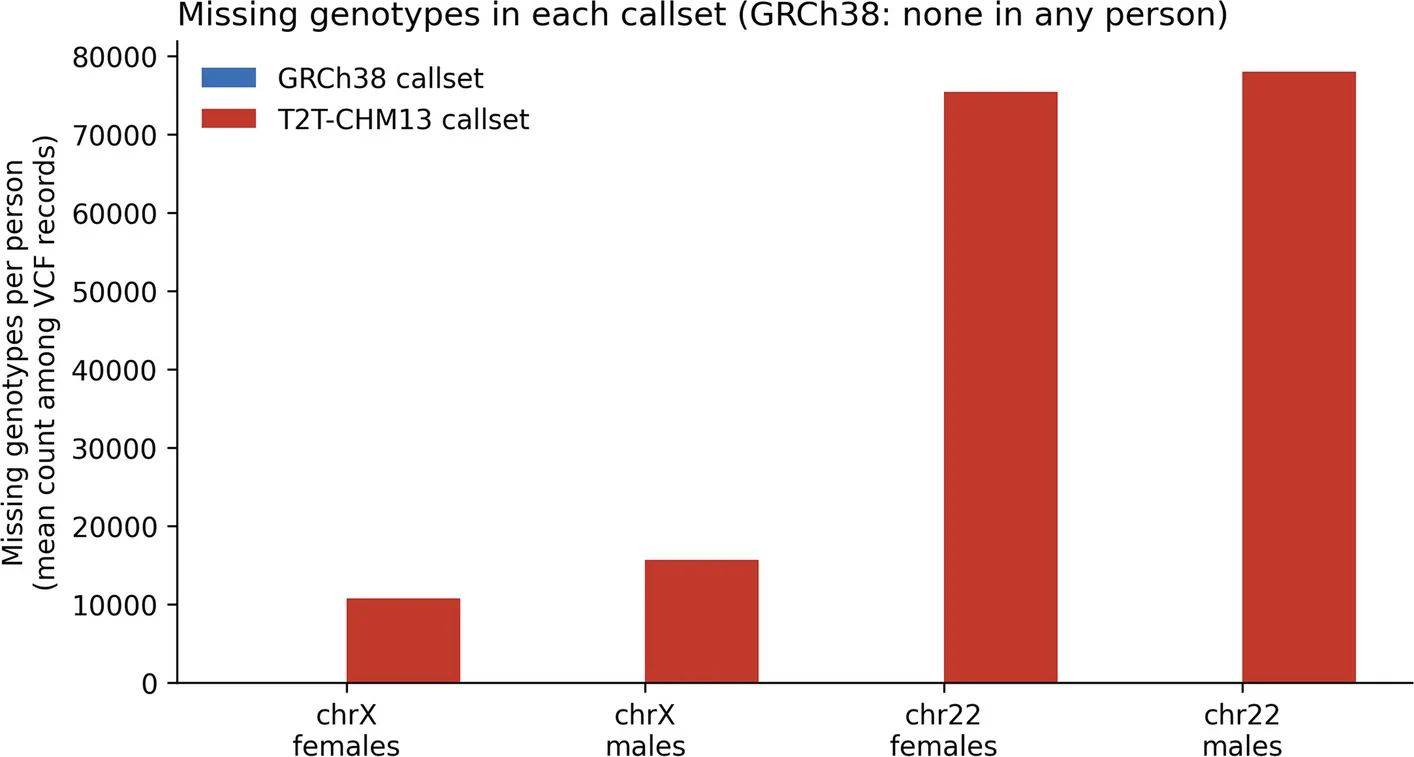
Mean missing genotypes per person among VCF records, by chromosome and sex. The GRCh38 callset has none

Chromosome 22 changes too. nHet rose by 13,873 per woman ([+ 13,714, + 14,029]) and 13,674 per man ([+ 13,510, + 13,833]), and nNonRefHom rose by 3,959 and 4,028. The heterozygous fraction rose from 0.6537 to 0.6867 in women (+ 0.0330) and from 0.6519 to 0.6837 in men (+ 0.0318). T2T has 75,391 missing genotypes per woman and 78,002 per man on chr22 (Figs. 3 and 4). The callsets therefore differ in site content and missingness on an autosome, in both sexes and by nearly the same amounts, as well as on chrX.

**Fig. 4:**
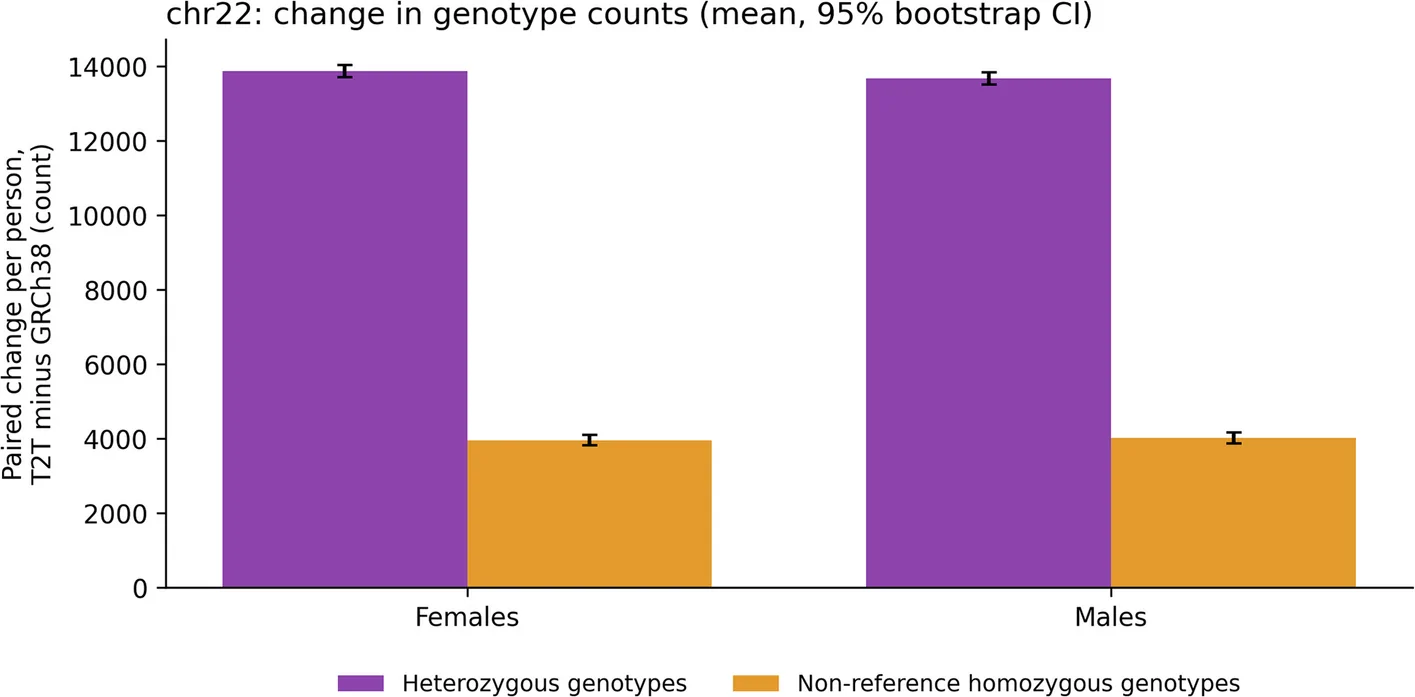
Paired change in chr22 genotype counts (T2T minus GRCh38) in women and men. Mean and 95% bootstrap CI

### chrX SNP lists share no coordinate strings

The first 500 chrX SNPs in VCF order (minor-allele frequency ≥ 0.01, call rate ≥ 0.95) on the two callsets share no chromosome-coordinate strings. Both lists lie in the pseudoautosomal region at the start of the chromosome, so they say nothing about non-PAR chrX. A coordinate is meaningful only on the assembly it was written against, so the lack of overlap says nothing about which loci are homologous, which are specific to one assembly, or where genotype calls agree. I did not lift variants between assemblies and did not measure concordance at allele-matched loci.

### Panel A: the male–female gap changes in both directions

In Panel A the gap *g* is negative under both callsets for all five models: women are classified better than men (Table 3; Fig. 5). Logistic regression, random forest and XGBoost widened the female advantage on T2T (ΔΔ = + 0.022, + 0.066 and + 0.042). The support vector machine and the perceptron narrowed it (ΔΔ = − 0.136 and − 0.031), and only the support vector machine remained significant after correction. In every model women’s balanced accuracy changed more than men’s. For logistic regression, random forest and XGBoost, accuracy rose on T2T, by 0.047, 0.071 and 0.050 in women and by 0.025, 0.005 and 0.009 in men. For the support vector machine and the perceptron, accuracy fell on T2T, by 0.190 and 0.072 in women and by 0.054 and 0.041 in men. The narrower gap for the support vector machine reflects a collapse in women’s accuracy to about that of men (0.4145 against 0.3994 on T2T) and is not an equalizing gain.

**Table 3:** Panel A balanced accuracy (BA) by sex and callset, and ΔΔ (20 splits)

Model	BA male, GRCh38	BA female, GRCh38	BA male, T2T	BA female, T2T	ΔΔ	95% CI	Holm *p* (panel)	Holm *p* (all 20)
Logistic Reg.	0.550	0.685	0.574	0.732	+ 0.022	[+ 0.008, + 0.037]	0.015	0.129
Random Forest	0.658	0.722	0.664	0.793	+ 0.066	[+ 0.048, + 0.083]	0.001	0.007
XGBoost	0.653	0.724	0.662	0.774	+ 0.042	[+ 0.023, + 0.060]	0.001	0.004
SVM	0.454	0.605	0.399	0.415	− 0.136	[− 0.152, − 0.122]	0.001	0.004
MLP	0.550	0.636	0.509	0.564	− 0.031	[− 0.067, + 0.007]	0.133	1.000

**Fig. 5:**
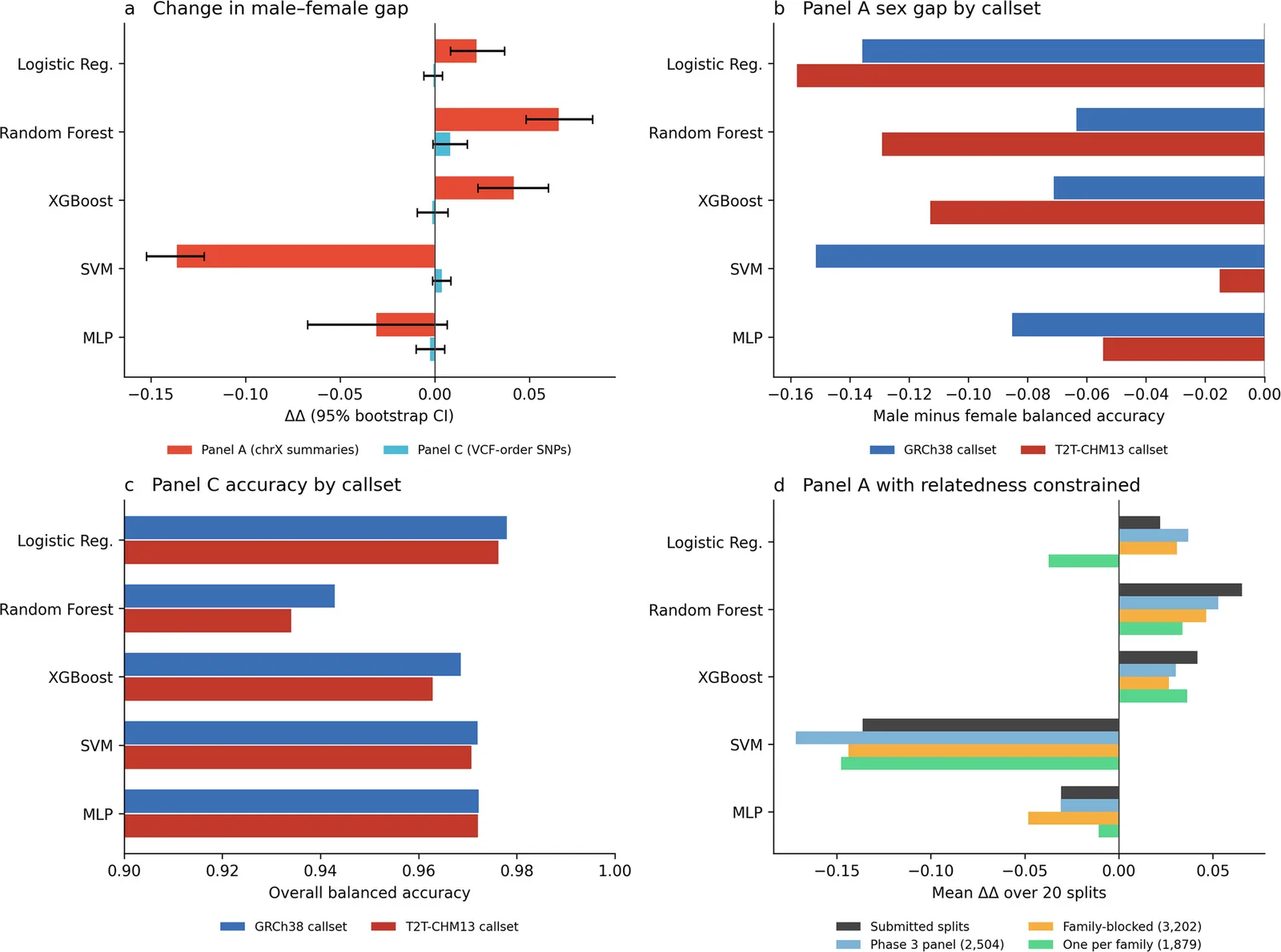
Panel battery. **a** ΔΔ for Panels A and C with 95% CI. **b** Panel A sex gap by callset. **c** Panel C accuracy by callset. **d** Panel A ΔΔ with relatedness constrained

Across the whole family of 20 tests, random forest, XGBoost and the support vector machine stay significant (Holm-adjusted *p* = 0.007, 0.004 and 0.004) and logistic regression does not (*p* = 0.13). The logistic regression, support vector machine and perceptron all used the same training-fold scaler, so the opposite sign for the support vector machine is a difference among model families that were scaled alike. Because the signs of the five models do not agree, no tier is assigned to Panel A.

### Panel A with relatedness constrained

I refit Panel A with the phase 3 panel, with family-blocked splits, and with one person per family (Table 4; Fig. 5d). On the phase 3 panel and under family-blocked splits, logistic regression, random forest and XGBoost kept a positive mean ΔΔ and the support vector machine kept a negative one. With one person per family, random forest (+ 0.034) and XGBoost (+ 0.036) stayed positive and the support vector machine stayed negative (− 0.148), but logistic regression turned negative (− 0.037, positive in 4 of 20 splits). For the linear and tree models, the range of ΔΔ across splits included zero in every analysis. Keeping families together did not remove the disagreement among models, and thinning to one person per family removed the logistic regression result.

**Table 4:** Panel A mean ΔΔ under relatedness constraints (20 splits)

Analysis	n	Logistic Reg	Random Forest	XGBoost	SVM	MLP
Phase 3 panel	2,504	+ 0.037	+ 0.053	+ 0.030	− 0.172	− 0.031
Family-blocked splits	3,202	+ 0.031	+ 0.046	+ 0.027	− 0.144	− 0.048
One person per family	1,879	− 0.037	+ 0.034	+ 0.036	− 0.148	− 0.011

Panel B saturates. Balanced accuracy exceeds 0.999 under both callsets, which leaves no room to measure a change in the gap.

### Panels C and D: VCF-order SNP controls

Panel C showed no change after correction (Table 5; Fig. 6; median ΔΔ = − 0.0007). Capping the T2T dosages at 2 left that conclusion unchanged: the five estimates moved by at most 0.004, all intervals still included zero, and no model was Holm-significant (median ΔΔ = + 0.0020; Table 5). In Panel D the estimates were small and of mixed sign (− 0.006 to + 0.013), and none was Holm-significant. The tier rule scored Panel D as suggestive on the strength of its median, which I no longer count as agreement. These results hold for the VCF-order selection rule only. I did not build random, LD-pruned or homologous panels. Three checks speak to the control itself and not to homology. Label permutation brought balanced accuracy to about 0.20, the chance level for five classes (Table 6). Leave-one-superpopulation-out validation lowered balanced accuracy to 0.73–0.80 without a consistent male–female gap (Table 7). Downsampling to exact sex balance (*n* = 2,150) stayed within 0.3 percentage points of the full-cohort accuracy (Table 8). None of these checks removes relatives from the training and test sets.

**Table 5:** Panel C ΔΔ (genome-wide common SNPs, VCF order)

Model	ΔΔ	95% CI	Holm *p*	ΔΔ, T2T dosage capped at 2	95% CI	Holm *p*
Logistic Reg.	− 0.0007	[− 0.006, + 0.004]	1.000	− 0.0018	[− 0.006, + 0.003]	1.000
Random Forest	+ 0.0081	[− 0.001, + 0.017]	0.520	+ 0.0066	[− 0.001, + 0.016]	0.713
XGBoost	− 0.0013	[− 0.009, + 0.007]	1.000	+ 0.0020	[− 0.006, + 0.010]	1.000
SVM	+ 0.0038	[− 0.001, + 0.009]	0.653	+ 0.0042	[− 0.001, + 0.009]	0.663
MLP	− 0.0024	[− 0.010, + 0.005]	1.000	− 0.0007	[− 0.007, + 0.006]	1.000

**Fig. 6:**
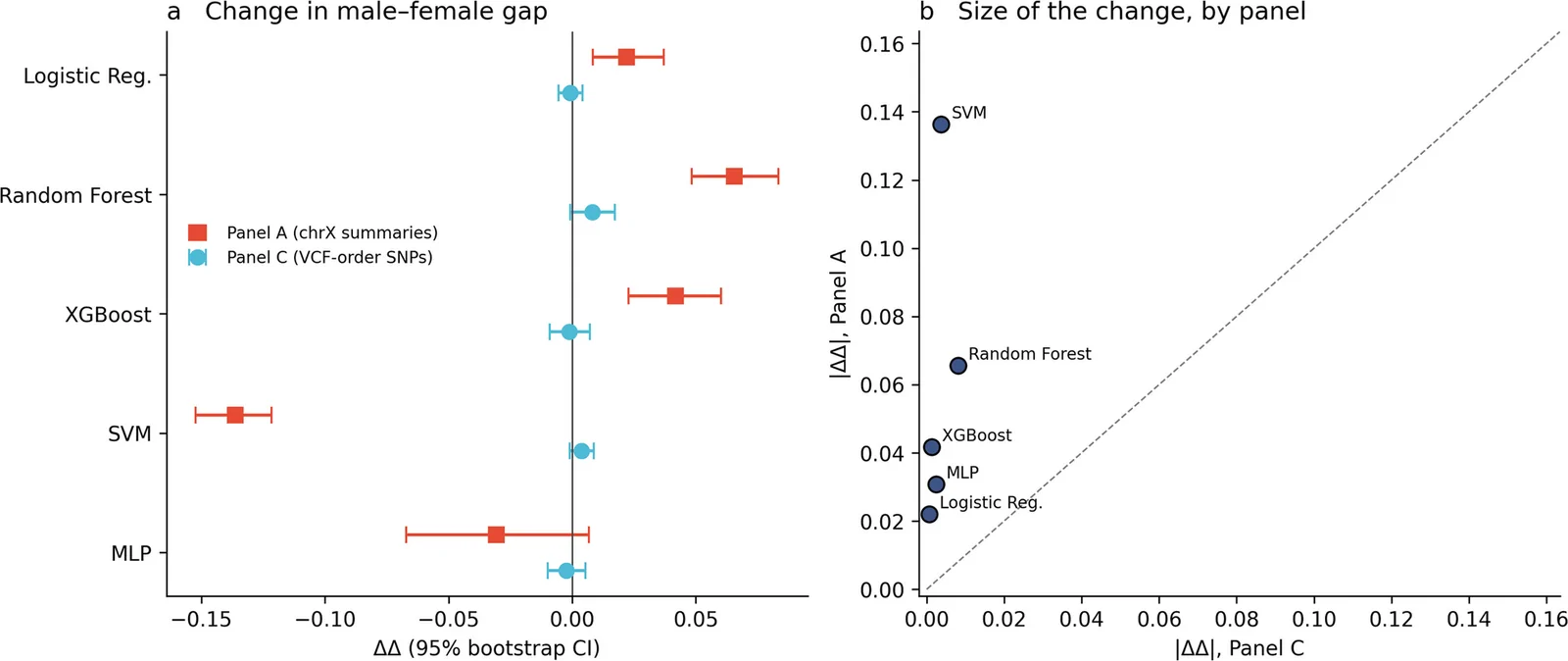
ΔΔ for Panel A and Panel C. **a** Estimates with 95% CI. **b** Absolute ΔΔ, Panel A against Panel C

**Table 6:** Label permutation (Panel C, selected models)

Callset	Model	True BA	Permuted BA (mean)
GRCh38	Logistic Reg.	0.980	0.204
GRCh38	Random Forest	0.936	0.209
T2T	Logistic Reg.	0.982	0.190
T2T	Random Forest	0.941	0.209

**Table 7:** Leave-one-superpopulation-out balanced accuracy (Panel C, selected conditions)

Callset	Model	Held out	Male BA	Female BA	Overall BA
GRCh38	Logistic Reg.	AFR	0.784	0.763	0.773
GRCh38	Logistic Reg.	EUR	0.797	0.800	0.799
T2T	Logistic Reg.	AFR	0.786	0.771	0.777
T2T	Logistic Reg.	EUR	0.800	0.800	0.800

**Table 8:** Sex-balance stress test (Panel C, exact parity, *n* = 2,150)

Callset	Model	Male BA	Female BA	Overall BA
GRCh38	Logistic Reg.	0.980	0.971	0.975
GRCh38	Random Forest	0.949	0.933	0.941
T2T	Logistic Reg.	0.986	0.985	0.985
T2T	Random Forest	0.923	0.943	0.934

## Discussion

The two public callsets give different chrX summaries for the same women. The heterozygous fraction is higher on the T2T callset in four superpopulations and lower in African-ancestry women, and the count data say why: non-reference homozygous genotypes disappear, and heterozygous genotypes do not appear. That is not the pattern a simple story of newly resolved sequence yielding extra heterozygous sites would predict, and I did not find it. I also cannot say that the pattern belongs to the assembly. The GRCh38 file is a phased panel whose stored tables show no missing genotypes, the T2T file is a recalibrated PASS callset with thousands, the T2T chrX file carries far more records per man, and chr22, which shares none of the chrX complications, shifts by nearly the same amount in women and men. Earlier work on the cost of incomplete or mishandled sex-chromosome references concerned alignment and variant discovery [7, 19]. The present data cannot add to that question, because assembly and workflow changed together. Isolating the assembly would take the same reads, one aligner, one caller and one filter set, run against both references.

The classifiers do not agree on what the change of callset does. Women were classified better than men under both callsets, and in all five models women’s accuracy changed more than men’s, upward or downward depending on whether the model improved or deteriorated on the T2T summaries. Random forest gained almost entirely in women (0.071 against 0.005). The support vector machine lost 0.190 in women and 0.054 in men, and its narrower gap is the result of a worse model for everyone. Neither number measures biological sex bias, and I would not read either as evidence that T2T-CHM13 creates or removes one. The six features are themselves built differently for the two sexes, since diploid and haploid genotypes enter them differently, and I did not check what the classifiers key on. Incomplete references can produce false sex differences in other measurements, as in metagenomic host-read filtering [20]. That is a different measurement from this one. Sex-aware quality control remains good practice for the reason given by Khramtsova and colleagues [21]: sex can interact with the way genotypes are produced.

The relatedness check shows how fragile the weaker results are. Family-blocked splits and the phase 3 panel left the signs of the tree models and the support vector machine in place. One person per family turned logistic regression negative, and a Holm correction over the whole family of 20 tests removed it. Random forest, XGBoost and the support vector machine are the results I would carry forward, and then only as results of this feature construction on these splits. The 20 splits share people, so the intervals describe stability across splits.

The VCF-order SNP panels are a reproducible control but not a representative one. They are not matched across assemblies, not LD-pruned and not random, and the absence of a change in them does not show that every autosomal classifier would be unaffected. A more convincing control would lift variants between assemblies, keep alleles that match at homologous loci, and draw LD-pruned panels repeatedly. Other limits are plain. Heterozygous fraction and the stored call rate are proportions among each file’s own records and not rates per callable non-PAR base. Reference-homozygous counts are absent from the stored tables, and recomputing them requires the VCFs, whose T2T chrX file alone is about 54 GB. Genotype concordance at lifted loci was not computed, and the extra genotypes were not localized to sequence that is new on T2T-CHM13. Panel D has the same dosage coding as Panel C and was not refit with capped values, and neither panel was refit with relatives separated. The five classifiers were treated as one family, and I did not choose a primary model after seeing the results.

For a laboratory changing reference, the practical advice is modest. Report which callset and workflow sit behind each reference. Report genotype counts alongside any ratio. Report each model and each sex separately, since a single summary hid opposite results here. Include controls that do not depend on the region under study, and state how they were chosen.

## Data Availability

The genomic callsets analyzed in this study are publicly available from the 1000 Genomes Project: GRCh38 NYGC high-coverage VCFs and T2T-CHM13v2.0 VCFs. Analysis code, processed intermediate results, and figure/table outputs supporting the findings are available at https://github.com/rstil2/reference-genome-sex-bias. No new human sequencing data were generated.
